# HIV-1 Induced Nuclear Factor I-B (NF-IB) Expression Negatively Regulates HIV-1 Replication through Interaction with the Long Terminal Repeat Region

**DOI:** 10.3390/v7020543

**Published:** 2015-02-05

**Authors:** Sai Vikram Vemula, Ravichandran Veerasamy, Viswanath Ragupathy, Santanu Biswas, Krishnakumar Devadas, Indira Hewlett

**Affiliations:** Laboratory of Molecular Virology, Center for Biologics Evaluation and Research, Food and Drug Administration, Bethesda, MD 20892, USA; E-Mails: veerasamy.ravichandra@nih.gov (V.R.); viswanath.ragupathy@fda.hhs.gov (V.R.); santanu.biswas@fda.hhs.gov (S.B.); krishnakumar.devadas@fda.hhs.gov (K.D.)

**Keywords:** Retrovirus, HIV-1, nuclear factors, latency, negative regulator, long terminal repeat

## Abstract

Background: Retroviruses rely on host factors for cell entry, replication, transcription, and other major steps during their life cycle. Human Immunodeficiency Virus-1 (HIV-1) is well known for utilizing a plethora of strategies to evade the host immune response, including the establishment of latent infection within a subpopulation of susceptible cells. HIV-1 also manipulates cellular factors in latently infected cells and persists for long periods of time, despite the presence of successful highly active antiretroviral therapy (HAART). Results: In this study we demonstrate that Nuclear Factor-IB (NF-IB) is induced during HIV-1 infection and its expression negatively impacts viral replication. During HIV-1 infection in peripheral blood mononuclear cells (PBMCs), and the T cell line, Jurkat or during induction of virus replication in latently infected cells, ACH2 and J1.1, we observed a time-dependent alteration in NF-IB expression pattern that correlated with HIV-1 viral expression. Using the Chip assay, we observed an association of NF-IB with the long terminal repeat region of HIV-1 (LTR) (-386 to -453 nt), and this association negatively correlated with HIV-1 transcription. Furthermore, knock-down of NF-IB levels in J1.1 cells resulted in an increase of HIV-1 levels. Knock-down of NF-IB levels in J-Lat-Tat-GFP (A1), (a Jurkat cell GFP reporter model for latent HIV-1 infection) resulted in an increase in GFP levels, indicating a potential negative regulatory role of NF-IB in HIV-1 replication. Conclusion: Overall, our results suggest that NF-IB may play a role in intrinsic antiretroviral defenses against HIV-1. These observations may offer new insights into the correlation of the latently infected host cell types and HIV-1, and help to define new therapeutic approaches for triggering the switch from latency to active replication thereby eliminating HIV-1 latent infection.

## 1. Introduction

Nuclear factor I (NF-I) is a family of transcription factors comprising four members (NF-IA, NF-IB, NF-IC, and NF-IX), each with distinct functions depending on the cell type and target promoter context [1]. Transcripts of each of the NF-I genes are differentially spliced, and NF-I recognition sequences are found in the sequences of many cellular and viral promoters, where they may act as activators or repressors of transcription [2].

A growing number of studies have shown the importance of NF-I proteins in several cellular and viral mechanisms. NF-I recognition sites have been shown to be present in many cellular and viral promoters/enhancers as well as in genomes of several viruses including adenovirus [3], BK virus (BKV) [4], human JC virus (JCV) [5], human papillomavirus (HPV) [6], herpes simplex virus 1 (HSV-1) [7], and cytomegalovirus (CMV) [8]. Even though the functional importance of these NF-I sites in regulating gene transcription is well established, their role in regulation of other viruses has not been well understood.

HIV-1 proviral gene expression is tightly regulated by an interplay of host and viral proteins with the regulatory elements present in the LTR sequence of the viral genome [2]. The HIV-1 LTR contains three discrete regions referred to as U3, R, and U5, and these contain four functional regions: the transactivation response (TAR) element, found within the R region (nt +1 to +60), the basal or core promoter (nt −78 to −1), a core enhancer region (nt −105 to −79), and a modulatory region (nt −454 to −104). The modulatory region contains the negative regulatory element (NRE) [2,9].

Key elements, such as the TATA, Sp1 and NF-kB region, that are present in the proximal part of the LTR (+1 to −112) have been extensively characterized [2]. However, transcription control elements present in the −112 to −459 modulatory region of the LTR have been poorly characterized. This region includes regulatory elements that can mediate transcription in many cell types, under a variety of growth and differentiation conditions [8]. One such regulatory element spanning the −385 to −362 region of the LTR, is directly adjacent to the NRE, has previously been foot printed using nuclear extracts from Jurkat cells and named as site A [9]. This core sequence, TGATTGGC, was shown to be the binding site for nuclear proteins present in a great variety of cell lines, such as Jurkat, HeLa, astrocytoma, oligodendroglioma and neuronal cells. Further, Schwartz *et al.* [9] have shown this region (−385 to −362) to be the binding site for nuclear protein and belonging to the nuclear factor I (NF-I) family. Interestingly, they observed an antagonistic role of this NF-I binding in the control of HIV-1 transcription in Jurkat cells.

Following the emergence of HIV-1 from the latent state and during the subsequent completion of the infection cycle, the host cell exhibits ordered changes in the expression of a subset of its genes, which shadow the well-known, ordered changes in the pattern of viral gene expression characteristic of the HIV-1 replication cycle [10]. Several studies have shown that treating host cells carrying HIV-1 provirus with different stimulants can re-activate the latent virus in these cells. We assumed that understanding of the differentially expressed NF-I protein levels during HIV-1 infection or reactivation of the latent virus into a replication cycle may provide potential new therapeutic approaches to eliminate viral infection.

## 2. Materials and Methods

### 2.1. Cells and Viruses

Jurkat, J-Lat-Tat-GFP-A1, ACH-2, and J1.1 cell lines were procured from the NIH AIDS Research and Reference Reagent Program (Rockville, MD, USA). PBMCs were isolated from three healthy HIV-seronegative donors obtained from the NIH blood bank by Ficoll-Hypaque separation (Sigma, St Louis, MO, USA). The cell lines and PBMCs were maintained in RPMI 1640 growth medium supplemented with 10% fetal bovine serum (FBS), 1 × penicillin/streptomycin, and 2 mM L-glutamine for 4 days in presence of 5 ug/mL phytohemagglutinin (PHA, Sigma) before the addition of 5 U/mL of human recombinant interleukin-2 (R&D systems, Minneapolis, MN, USA). The HIV-1 IIIB strain used in this study was obtained from the AIDS Research and Reference Reagent Program and propagated in Jurkat cells and PBMCs. Culture medium from HIV-1 infected cultures was collected every 3 days, clarified by centrifugation at 1500 RPM for 5 min, and quantitated for p24 antigen using an in-house HIV-1 europium nanoparticle P24 ELISA.

### 2.2. Infection of PBMCs and Jurkat Cells with HIV-1 Virus and Reactivation of Latently Infected Cells

The expression of NF-IB during HIV-1 infection was tested in HIV-1 infected Jurkat cells and PBMCs. PBMCs and Jurkat cells were infected with HIV-1 III-B strain using 5 ng equivalent of p24/10^6^ cells) in RPMI 1640 growth medium for 3 h at 37 °C. Infected cells were washed once with PBS and resuspended in RPMI 1640 growth media. At various time-pointsfollowing HIV-1 infection, cell pellets and supernatants were collected and stored at −80 °C to measure the expression of HIV-1 gag and NF-IB gene and proteins respectively. Reactivation of latency by PMA treatment: ACH-2, and J1.1 cells were reactivated by treatment with 10 ng/mL of phorbol myristyl acetate (PMA) for 30 min at 37 °C [10]. Following PMA treatment cells were washed twice in PBS. On days 1, 2, and 3 post activation cell pellets and supernatants were collected and stored at −80 °C.

### 2.3. Nuclear Extract Preparation

Nuclear and cytosolic fractions were prepared from infected cells using a nuclear extraction kit (Active Motif, Carlsbad, CA, USA). Briefly, the cells were washed once using 1 × PBS and then re-suspended in an ice cold hypotonic buffer (10 mM HEPES, pH 7.9, 10 mM KCl, 0.1 mM EDTA, 0.1 mM EGTA, 1 mM dithiothreitol (DTT), and 0.5 mM phenylmethylsulfonyl fluoride (PMSF) and incubated on ice for 15 min. After the addition of Nonidet P-40 (final concentration-0.5%), cells were vortexed for 10 s and centrifuged for 30 s at 14,000 g at 4 °C. The supernatant (containing the cytosolic fraction) and pellets (containing the nuclear fraction) were collected. Nuclear pellets were resuspended in a buffer containing 20 mM HEPES, pH 7.9, 20% glycerol, 0.4 M NaCl, 1 mM EDTA, 1 mM EGTA, 1 mM DTT, 1 mM PMSF, and Complete^®^ Mini Protease Inhibitor Cocktail (Roche Diagnostics GmbH, Mannheim, Germany) and incubated for 60 min on a rocking platform at 4 °C. After a brief vortex, nuclear fractions were separated by centrifugation at 14,000×g for 10 min at 4 °C. Nuclear proteins in the supernatant were collected and stored at −80 °C until used. For total cell lysate, cells were lysed with RIPA buffer (Santa Cruz Biotechnology, Santa Cruz, CA, USA) supplemented with 1 mM EDTA, 1 mM EGTA, 1 mM DTT, 1 mM PMSF, Complete^®^ Mini Protease Inhibitor Cocktail. Protein concentration was measured using a BCA protein assay reagent kit (Thermo Scientific, Rockford, IL, USA).

### 2.4. Western Blotting

Equal amounts of protein separated on a NuPAGE 4%–12% Bis-Tris gel (Invitrogen, Grand Island, NY, USA) were transferred on to a 0.45 µm pore size polyvinylidene fluoride (PVDF) membrane (Invitrogen). Membranes were blocked for 1 h with 5% non-fat dry milk. Blots were then probed using antibodies against NF-IA (rabbit polyclonal from Active Motif), NF-IB (rabbit polyclonal from Active Motif), β-actin (clone AC-74 from Sigma), gp41 (goat polyclonal from Thermo Fisher Scientific, Rockford, IL, USA).

The bound antibodies were detected using appropriate secondary antibodies conjugated with horseradish peroxidase (Santa Cruz biotechnology). Blots were developed and visualized by Western blotting Luminol reagent (Santa Cruz biotechnology) according to the manufacturer instructions. Quantification of signals from Western blots was done using Image J software, version.

### 2.5. LTR Pull-Down Assay

The HIV-1 LTR region was amplified using a full length HIV-1 IIIB clone as the template. A 456 bp fragment amplified from GAPDH coding region was used as a negative control. Both non-specific biotinylated DNA (bio-NS), and biotinylated LTR-probes (bio-LTR) were added with streptavidin-agarose in a binding buffer (10 mM HEPES, pH 7.8; supplemented with 1 mM EDTA, 0.5 mM PMSF Complete^®^ mini protease inhibitor cocktail (Roche Diagnostics GmbH) and gently rotated for 2 h at 4 °C. Unbound biotinylated probes were removed by washing with binding buffer, and bound Biotin-streptavidin-agarose beads (agar-strep-bio-NS or agar-strep-bio-LTR) were made into 50% slurry using binding buffer.

Nuclear extracts were pre-cleared by gently rotating with 10% slurry of NS-Bio-streptavidin-agarose beads for 1 h at 4 °C. After centrifugation, the supernatants (pre-cleared nuclear extracts) were added to equal volumes of protein binding buffer containing agar-strep-bio-NS or agar-strep-bio-LTR. After 2 h at 4 °C with gentle rotation, the agar-strep-bio-NS or agar-strep-bio-LTR were pelleted by centrifugation, and washed three times with wash buffer (10 mM HEPES, pH 7.8, 0.1% NP-40, 0.5% glycerol, Complete^®^ mini protease inhibitor cocktail (Roche Diagnostics). Proteins that bound to the agar-strep-bio-NS or agar-strep-bio-LTR were eluted by boiling for 3 min in Laemmli protein loading buffer and then resolved on a 4%–12% NuPAGE Bis-Tris gel. The immunoblotting was performed as described earlier.

### 2.6. Chromatin Immunoprecipitation (Chip) Assay

Chip assay was performed using the Chip-IT Express enzymatic kit (Active Motif) according to the manufacturer’s instructions. Briefly, the cells were fixed using 1% formaldehyde, lysed and then the cellular DNA was sheared using a Enzymatic shearing cocktail. One-tenth of the total cell lysate was used for total genomic DNA as “input DNA” control. Immunoprecipitation was performed overnight in cold room with 2 µg of NF-1B antibody (Active Motif) or β-actin (Sigma) and mouse IgG (Active Motif) antibodies as negative controls. Protein A/G was added and incubated at 4 °C for 1 h. Beads were then washed three times in Chip buffer, after which elution of the digested chromatin, reversion of cross-linking, and proteinase K treatment was performed. The eluted immunoprecipitated DNA and the sample of Chip input DNA were purified with the QIAquick PCR Purification Kit and then subjected to quantitative PCR (qPCR) by employing NF-1B site flanking primers: LTR Chip primer fwd (5'-TGGAAGGGCTAATTCACTCC-3'), LTR Chip primer rev (5'-GTAGTTCTGCCAATCAGGGAAGTAGC-3'). GAPDH was amplified using primers (FWD: 5’-GACAGTCAGCCGCATCTTCT; and REV: 5’- TTAAAAGCAGCCCTGGTGAC) and used to normalize the amount of input material.

### 2.7. Real-Time RT- PCR

Total RNA was extracted from the cells using the RNAzol-RT kit (Molecular Research Center, Inc., Cincinnati, OH, USA) according to the manufacturerss instructions. RNA concentration was measured using a Nanodrop spectrophotometer. First-strand cDNA was synthesized from 5 µg of total RNA using a SuperScript III first-strand synthesis kit (Invitrogen). Quantitation of NF-IA, NF-IB, NF-IC, and NF-IX, HIV-1 gag, and GAPDH were done using the following primers (NFI-A, Forward-CAGCCAAGTGACGCTGACA, Reverse-CCTCATTGCTCCTGGACTCAT; NF-1B, Forward-GCCACAATGATCCTGCCAAGAA, Reverse-GGTGGAGAAGACAGAGACCTCTGA; NFI-C, Forward-GGACAGGGATGGGCTCTG, Reverse-CGTTCTTCTGAGGCCAGTGC; NFI-X, Forward-CCACTGCCCAACGGACACTT, Reverse-CCGGGATAGAACACGTCATCA; HIV-1 gag Forward-CTAGGAACGATTCGCAGTTAATCCT, Reverse-CTCTATTGTGTGCATCAAAGGATAG; GAPDH Forward-GACAGTCAGCCGCATCTTCT, Reverse-TTAAAAGCAGCCCTGGTGAC).

Real-time PCR was performed on ABI Prism 7500 instrument (Applied Biosystems, Foster City, CA, USA) with a quantitect SYBR green kit (Qiagen, Valencia, CA, USA). The expression levels were normalized against glyceraldehyde-3-phosphate dehydrogenase (GAPDH).

### 2.8. Knock-Down of NF-IB Expression Using Lentivirus-Based shRNA Transduction

NF-IB level was knocked down in Jurkat, J1.1, and ACH-2 cells using MISSION shRNA lentiviral particles (Sigma). Briefly, cells (1.0 × 10^5^) were transduced with 1.0 × 10^5^ TU Lentiviral particles targeting human NF-1B (shNF-IB). As negative controls, cells were transduced with the MISSION pLKO.1-puro Non-Target shRNA Control Transduction Particles (sh-Control) that contain an shRNA insert that does not target any human gene. Three days post-infection, the cell media were supplemented with puromycin (10 ug/mL), and 2 weeks after selection, cells were used for the study. Knockdown of NF-1B expression in the resultant cell lines was confirmed by Western blot analysis. sh-Control or sh-NF-IB-selected Jurkat cells were infected with HIV-1 (III-B strain; 5 ng equivalent of p24/mL) and incubated for 10 days. J-Lat-Tat-GFP-A1 cells (1.0 × 10^5^) were infected with 1.0 × 10^5^ TU lentiviral particles (shNF-IB or sh-control). Three days post-infection, the media was supplemented with puromycin (10 µg/mL), and 2 weeks after selection, cells were used for the study. Jurkat cells were used to normalize cellular autofluorescence. Cells were washed in PBS and fixed in 1% paraformaldehyde. GFP fluorescence was measured using a FACScan flow cytometer (Becton Dickinson, Mountain View, CA, USA). In another set of experiments, the fluorescence from lysed cells was measured using GFP ELISA kit (Cell Biolabs. Inc., San Diego, CA, USA).

### 2.9. Statistical Analysis

The Students *t*-test was used for calculation of significance. The significance was set at *p* < 0.05.

## 3. Results

### 3.1. HIV-1 Infection Results in Upregulation of NF-1B Expression

**Figure 1:**
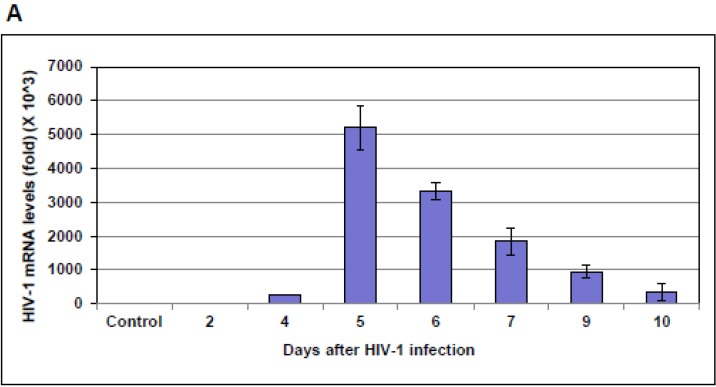

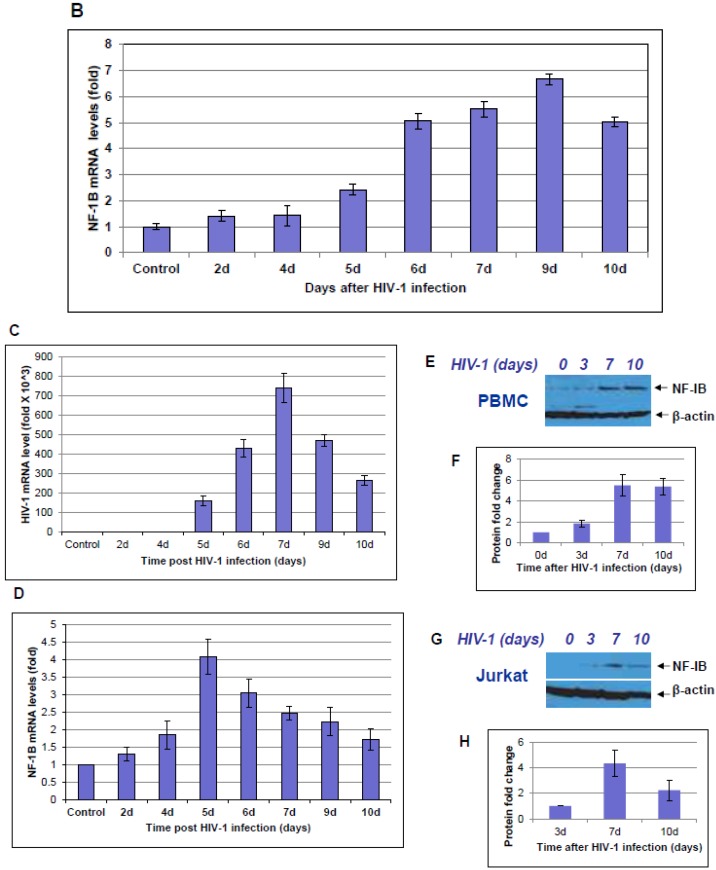
HIV-1 RNA expression level following HIV-1 infection. Peripheral blood mononuclear cells (PBMCs) from three different donors (**C**), and Jurkat cells (**A**) were infected with HIV-1 (III-B strain; 5 ng equivalent of p24/10^6^cells). At the indicated time points post-infection, 1 × 10^6^ cells were used to measure HIV-1 gag gene expression levels using real-time quantitative RT-PCR. NF-IB gene expression levels were also measured from the same PBMCs (**D**), and Jurkat cell samples (**B**). The data were normalized to glyceraldehyde-3-phosphate dehydrogenase (GAPDH levels) levels and represent the mean *±* standard error of the mean (SEM) from three experiments*.* The HIV-1 infected PBMCs (**E**) and Jurkat cells (**G**) were lyzed and the NF-IB protein level was measured using Western blot. Beta-actin was used as an internal control. Quantification of signals from Western blots was done using Image J software (1F; PBMCs and 1H; jurkat cells). Fold change is relative to 0 d time point. Data are averages of Western blot quantifications ± SEM. Representative Western blots are shown.

HIV-1 infection of PBMCs from three different donors showed a peak of virus production around six to seven days post-HIV-1 infection, while in Jurkat cells HIV-1 infection peaked at five days post-infection, as measured by the HIV-1 gag expression using real-time qPCR method (Figure 1A,C). We observed a consistent change in NF-IB level that was proportional to the HIV-1 infection. Further, we investigated the importance of NF-I family members, NF-IA, NF-IB, NF-IC, and NF-IX, on HIV-1 transcription by knocking-down the expression of individual NF-I members and infecting with HIV-1, and found that only the reduction of NF-IB levels caused a significant change in HIV-1 transcription (Figure S2). We therefore focused on the role of NF-IB in HIV-1 infection. We measured both NF-IB gene and protein expression levels in HIV-1 infected PBMCs and Jurkat cells. A fivefold increase in NF-IB levels was observed in both PBMCs and Jurkat cells (Figure 1B,D). Further, a gradual increase in NF-IB protein expression during HIV-1 infection was observed by Western blot using the anti-NF-IB antibody (Figure 1E).

**Figure 2:**
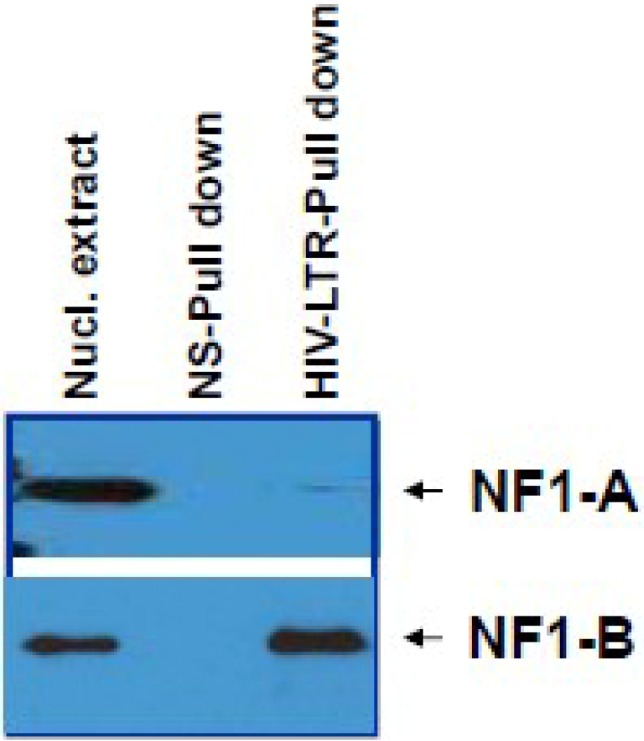
Specificity of NF-IB to HIV-1 LTR. HIV-1-infected Jurkat cells were harvested on day 5 post-infection. Nuclear extracts were prepared as descried under materials and methods and used for the long terminal repeat (LTR) pull-down experiments. To check the specificity of the NF-IB association, both non-specific (biotinylated GAPDH DNA region coupled with streptavidin agarose), and LTR HIV-1 (biotinylated LTR DNA coupled with streptavidin agarose) were used for the pull-down experiments. The bound proteins, along with the control nuclear extracts, were detected using NF-IA or NF-IB antibodies.

Even though we observed a change in expression profile of NF-I family members (NF-IA, NF-IB, NF-IC, and NF-IX) in HIV-1 infected cells, only increase in NF-IB expression influenced transcription of HIV-1 (Figure S1). HIV-1 LTR pull-down experiments using nuclear extracts from HIV-1 infected Jurkat showed specificity for NF-IB, but not for NF-IA (Figure 2).

### 3.2. Reactivation of Latently Infected Cells with PMA Treatment Results in an Increase in NF-1B Expression

**Figure 3:**
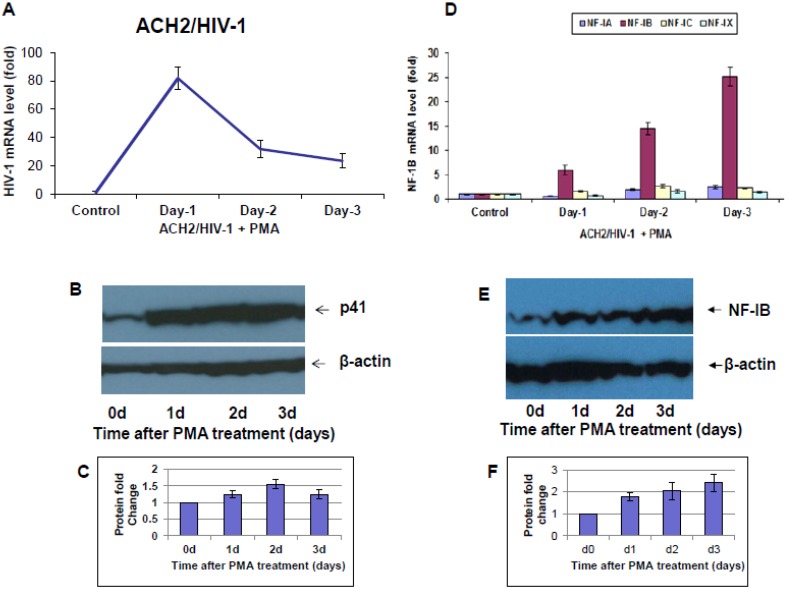
HIV-1 and NF-IB levels during re-activation of ACH2/HIV-1 cells. ACH-2 cells were stimulated with phorbol 12- myristate 13-acetate (PMA) for four days. At the indicated time point, cells were harvested to measure HIV-1 (**A**,**B**), and NF-IB (**D**,**E**) levels by both qRT-PCR and Western blotting, respectively. The data in Figure 3A,D were normalized to GAPDH levels and represent the mean *±* SEM from three experiments*.* (**C**,**F**). Quantification of signals from Western blots was done using Image J software. Fold change is relative to the 0 d time point. Data are averages of Western blot quantifications ± SEM. Representative Western blots are shown.

Several studies have shown that treating host cells carrying HIV-1 provirus with different stimulants can re-activate the latent virus in these cells. For example, latent cells can be re-activated *in vitro* using inducing agents such as phorbol myristyl acetate (PMA), which can induce replication in latent cells. In our studies, we observed that induction of ACH2 cells with PMA resulted in a substantial increase in HIV-1 RNA levels on day 1, and this higher level rapidly decreased on day 2, and continued to decline until three days post-PMA treatment (Figure 3A). In addition, Western blot performed on the same experimental samples using anti-HIV-1 41 antibody showed an increase in HIV-1 gp41 protein expression at day one, and continued for up to three days post-PMA induction (Figure 3B). Further, to understand if reactivation could cause any change in NF-I levels, we measured the expression of NF-I family members during PMA induction, and found that only NF-IB expression levels showed a time-dependent increase for up to three days, and decrease on day 4. Significantly, on day 3 post-PMA induction, we observed a 25-fold increase in NF-IB levels (Figure 3D). Western blot of NF-IB protein levels measured using anti-NF-IB during the PMA induction also showed an orderly increase of protein levels for up to 4 days (Figure 3E).

**Figure 4:**
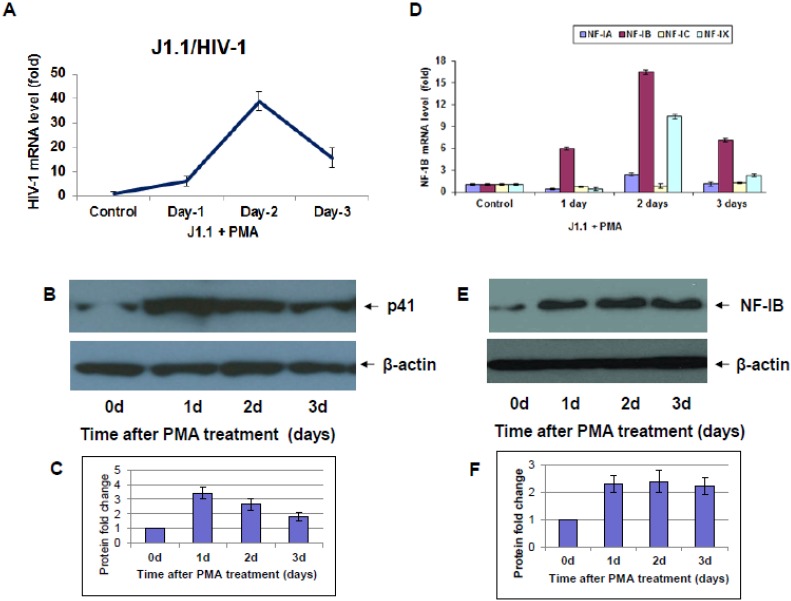
HIV-1 and NF-IB levels in J1.1 cells following PMA activation. J1.1 cells were stimulated with PMA and at indicated time points, cells were harvested to measure HIV-1 (**A**,**B**), and NF-IB (**D**,**E**) levels. The data in Figure 4A,D were normalized to GAPDH levels and represent the mean *±* SEM from three experiments*.* (**C**,**F**) Quantification of signals from Western blots was done using Image J software. Fold change is relative to the 0 d time point. Data are averages of Western blot quantifications ± SEM. Representative Western blots are shown.

Similar to ACH2 cells, reactivation of latent J1.1 cells showed an increase in HIV-1 infection after PMA induction; however, the peak for HIV-1 reactivation was 2-days post-PMA induction (Figure 4A,B). Further, J1.1 cells showed an increase in NF-IB level 2-days after PMA induction, and levels dropped on day 3 and day 4 (Figure 4D,E). Interestingly, a 38-fold increase in HIV-1 levels was observed after PMA induction concomitant with a 17-fold increase in NF-IB gene levels. However, in J1.1 cells, during the peak of reactivation (2-days), NF-IX showed about 10-fold increase in expression level (Figure 4D). In a separate experiment, we reactivated latent ACH2, and J1.1 cells using a different stimulant, TNF-α, and found similar increases in NF-IB levels following HIV-1 activation (data not shown).

### 3.3. HIV-1 Induced NF-1B Binds to HIV-1 LTR

**Figure 5:**
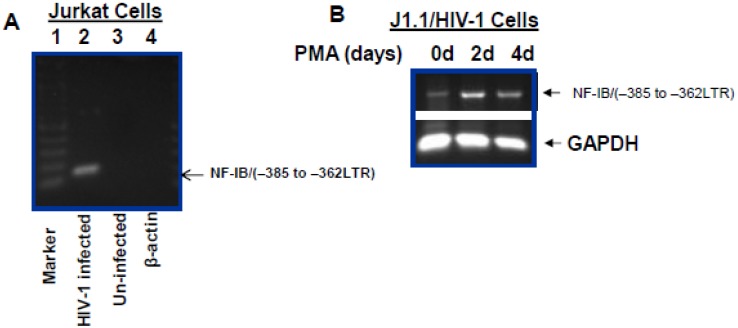
Association of NF-IB with the HIV-1 LTR. Chip assay was performed to see the *in vivo* association of NF-IB with the LTR in control and HIV-1 infected Jurkat cells (**A**); lane 1, 100 bp DNA marker; lane 2, samples from NF-IB antibody pull-down (HIV-1 infected); lane 3, samples from NF-IB antibody pull-down (Un-infected); lane 4, samples using β-actin antibody pull-down (HIV-1 infected). The latently infected cells, J1.1, were stimulated with PMA for four days, and Chip assay was performed on un-stimulated cells and PMA stimulated cells (**B**). GAPDH was amplified to show as an input control. The data in Figure 5A,B is representative of three independent experiments*.*

Based on published reports that HIV-1 LTR (–385 to –362) contains a binding site for NF-I, we investigated binding of NF-IB protein with the LTR. A Chip assay using NF-IB antibody was performed. As shown in Figure 5A, NF-IB bound to the HIV-1 LTR in HIV-1 infected Jurkat cells. To test the hypothesis that NF-1B induced following HIV-1 infection binds to the LTR and restricts transcription we conducted Chip assay in un-stimulated and PMA stimulated J1.1 cells. Interestingly, even though NF-IB was bound to the HIV-1 LTR in un-stimulated cells, PMA stimulated cells showed an increase in binding, proportional to NF-IB expression levels (Figure 5B).

### 3.4. Lentiviral-Mediated Silencing of NF-1B Expression Enhances HIV-1 Replication

**Figure 6:**
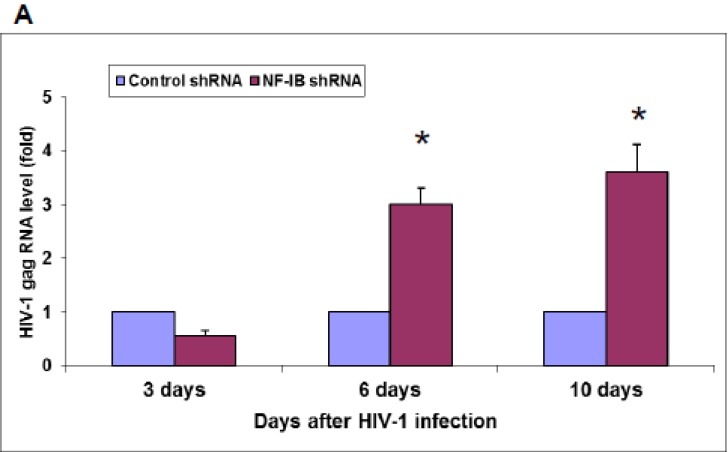

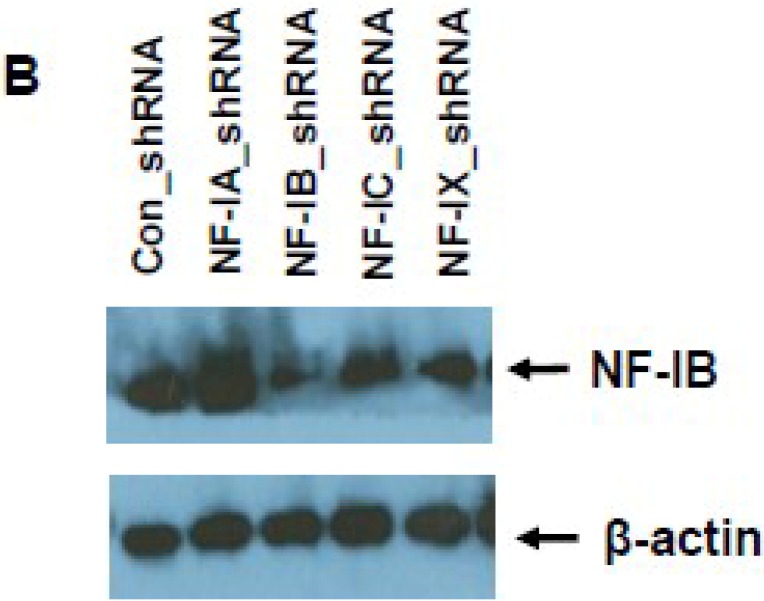
(**A**) HIV-1 infection in NF-IB knock-down PBMCs. shNF-IB or sh-control selected PBMCs were infected with HIV-1 for 10 days. On day 3, 6, and 10 post-HIV-1 infection, cells were tested for the HIV-1 gag levels using quantitative PCR. The data represent the mean *±* SEM from three experiments*.* (**B**). Knock-down of NF-I family members in PBMCs. PBMCs were used to knock-down NF-I family members expression levels using MISSION Lentiviral Transduction Particles. Reduction in the NF-IB protein expression was measured by Western blot. Beta-actin was used as internal control. * *p* ≤ 0.05 compared to control shRNA treated cells. The data presented are a relative fold change compared to control shRNA treated cells at each time point.

To further understand the role of NF-IB in HIV-1 transcription, we used a lentiviral mediated shRNA system, targeting NF-IB gene to knock-down NF-IB expression in PBMCs. Using this system, we were able to knock-down up to 80% of the *in vivo* NF-IB expression. HIV-1 infection of cells in which NF-1B expression was knockeddown resulted in a decrease in HIV-1 transcription at three days post-infection; however, at days 6 and 10 post-infection, the HIV-1 RNA (gag) level was increased compared to the control-shRNA treated cells (Figure 6).

**Figure 7:**
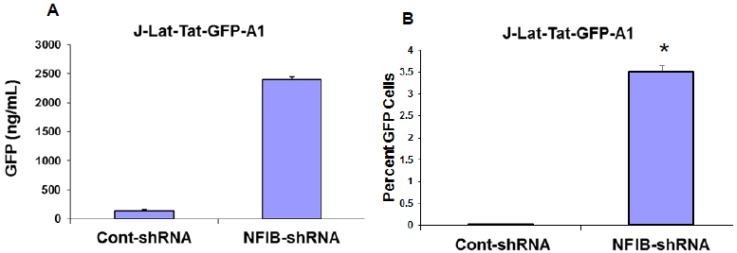

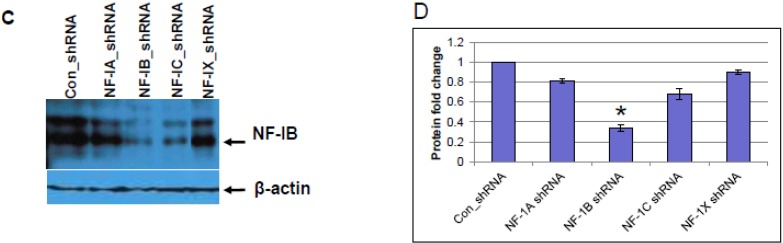
Knock-down of NF-IB in J-Lat-Tat-GFP cells. J-Lat-Tat-GFP-A1 cells were used to knock-down NF-IB level using MISSION lentiviral transduction particles two weeks after selection for the shNF-IB, or control-shRNA cells, the GFP fluorescence was measured using a FACScan flow cytometer (Becton Dickinson) (**A**). In another set of experiments, the fluorescence from the lysed cells was measured using GFP ELISA kit (Cell Biolabs Inc.) (**B**). The data were normalized to GAPDH levels and represent the mean *±* SEM from three experiments*.* Reduction in the NF-IB protein expression was measured by Western blot using NF-I knocked-down cells. Beta-actin was used as internal control (**C**). (**D**) Quantification of signals from Western blots was done using Image J software. Fold change is relative to control shRNA knock down cells. Data are averages of Western blot quantifications ± SEM. Representative Western blots are shown. * *p* ≤ 0.05 compared to control shRNA treated cells.

Using the same lentiviral system, we also knocked-down expression of NF-IB in J-Lat-Tat-GFP-A1 cells, a Jurkat cell GFP reporter model for latent HIV infection. Reduction of NF-IB levels in J-Lat-Tat-GFP-A1 cells resulted in an increase of GFP levels, as measured by both FACS and ELISA methods (Figure 7A–C). These results further substantiate the negative regulatory role of NF-IB on HIV-1 transcription.

## 4. Discussion

The persistence of latently infected viral reservoirs, despite prolonged highly active antiviral retroviral therapy (HAART), represents a major obstacle to the eradication of HIV-1 in infected patients [11]. Latently infected cells contain replication-competent integrated HIV-1 genomes that are blocked at the transcriptional level, resulting in the absence of viral protein expression [12,13].

During *de novo* HIV-1 infection, or reactivation of latently infected cells, various host cell genes are differentially expressed, most of which play an important role in regulating HIV-1 replication [10]. Here, we observed a marked difference in NF-IB expression during HIV-1 infection, or reactivation of latently infected cells. Further understanding of NF1-B modulation may offer interesting and new insights into viral pathogenesis and latency. Because NFI family members are shown to either repress or activate cellular and viral genes, it was not evident whether the up-regulation of NF-IB during HIV-1 infection was favorable for the virus or cell. Hence we studied the role of NF-IB on HIV-1 transcription, and observed a novel mechanism of negative regulation of HIV-1 transcription by NF-IB, which is induced by HIV-1 infection.

The NRE region of the HIV-1 LTR has been reported to down-regulate LTR-directed HIV gene expression [2,9]. However, the mechanism for down regulation is not well understood. Since recent data have highlighted the importance of the U3 region of the LTR in determining the pathogenicity of HIV-1 [9], studies concerning the nature and role of cellular transcription factors that interact with the LTR continue to be essential to our understanding of viral pathogenesis. Our results show induction of NF-IB protein in HIV-1 infection, and from our Chip experiment results it is apparent that HIV-1 induced NF-IB binds to the NRE region of the HIV-1 LTR (–385 to –362).

HIV-1 is known to manipulate host gene expression in favor of its replication and existence. For example, from our current study, the observed induction of NF-IB during HIV-1 infection and reactivation of latent infection, and its subsequent association with the NRE region of HIV-1 LTR suggests a negative feedback regulation of HIV-1 transcription through NF-IB. To support our hypothesis, in a lone available published result regarding the association of NF-I with LTR, it was demonstrated that the association of NF-I with TGATTGGC region of HIV-1 LTR resulted in antagonistic effect in the control of basal transcription of the HIV-1 genome in Jurkat cells [9].

Several studies have described changes in certain cellular genes during HIV-1 infection. While many studies provide insights into the extensive effects of specific viral proteins on acute HIV-1 infection, changes in gene expression that may accompany HIV-1 infection and subsequent transition of the acute phase into a virologically latent phase have not been fully understood. It is believed that after the entry into the cell, HIV-1 establishes efficient control of viral replication by manipulating the cellular machinery. Based on our observation, we speculate that the induction of NF-IB during HIV-1 infection is to contain viral transcription through binding with the NRE region of the HIV-1 LTR.

Interestingly, pre-existence of the association of NF-IB with the NRE region of the HIV-1 LTR in latently infected (un-stimulated) cells, observed in our Chip experiment, further substantiates our notion that NF-IB acts as a negative regulator. As the reactivation of latently infected cells by external stimuli activates HIV-1 transcription, which in turn induces the expression of NF-IB resulting in increased association with the LTR. Subsequently HIV-1 transcription level is diminished to near normal levels; ironically, as HIV-1 transcription decreased, the NF-IB levels return to background, normal levels. Therefore, we observed a direct correlation between HIV-1 infection and NF-IB induction, and an inverse correlation between the NF-IB association with the LTR and HIV-1 transcription. Consistent with these results, we observed an increase in HIV-1 transcription when NF-IB was knocked-down by siRNA treatment in latent infected cells, confirming the negative regulatory role of NF-IB on HIV-1 transcription.

In a separate study depicting the relationship between HIV-1 infection and NF-IB expression, Sheeter *et al.* [14] proposed a negative role of NF-IB on cell surface CD4 expression. For optimal viral production, HIV-1 infection requires surface CD4 receptor down-modulation. On the basis of their findings, they speculated that NF-1B may be a cellular factor that HIV-1 induces to assist in the down-modulation of CD4, thereby restricting the new viral entry into the cell. Taken together, HIV-1-induced NF-IB expression may have a dual role in containing HIV-1 transcription: (1) by binding with the NRE region of the HIV-1 LTR to contain viral transcription; and (2) by down-modulating surface expression of CD4 to sustain viral infection. These findings suggest that NF-IB is a cellular antiviral response triggered by HIV-1 to contain its infection.

NFI consensus binding sites have been found on many cellular gene promoters, and association of NFI proteins with these promoters has been proposed to play a role in the determination of gene expression [1]. Although further work will be required to clarify the correlation between NF-IB expression and HIV-1 transcription, data presented here conclusively demonstrates negative feedback regulation of HIV-1 transcription through the induction of NF-IB. In addition to binding to the HIV-1 LTR, HIV-1 induced NF-IB could also bind with the cellular gene promoters thereby altering the cellular mechanisms. The existence of the NF-I binding site on the various viral promoters raises the possibility that HIV-1 induced NF-IB could have effect on the transcription of other viruses, thereby regulating co-infections. The effect of HIV-1 induced NF-IB on the activation/inhibition of cellular and viral genes through binding on to the promoter remain open questions for future studies.

## 5. Conclusions

In summary, our data demonstrate the importance of NF-IB in HIV-1 transcription, and in conjunction with other transcription factors, NF-IB should be considered as an important transcription factors involved in the pathophysiology of the HIV-1 infection.

## Supplementary Files

Supplementary File 1
