# Supplementary material for: HIV-1 Induced Nuclear Factor I-B (NF-IB) Expression Negatively Regulates HIV-1 Replication through Interaction with the Long Terminal Repeat Region
Source: Viruses. 2015 Feb 5;7(2):543–58. doi: 10.3390/v7020543 (PMC4353903; doi:10.3390/v7020543)
Supplement: Supplementary File 1 [file viruses-07-00543-s001.docx]

**Supplementary Materials**

*
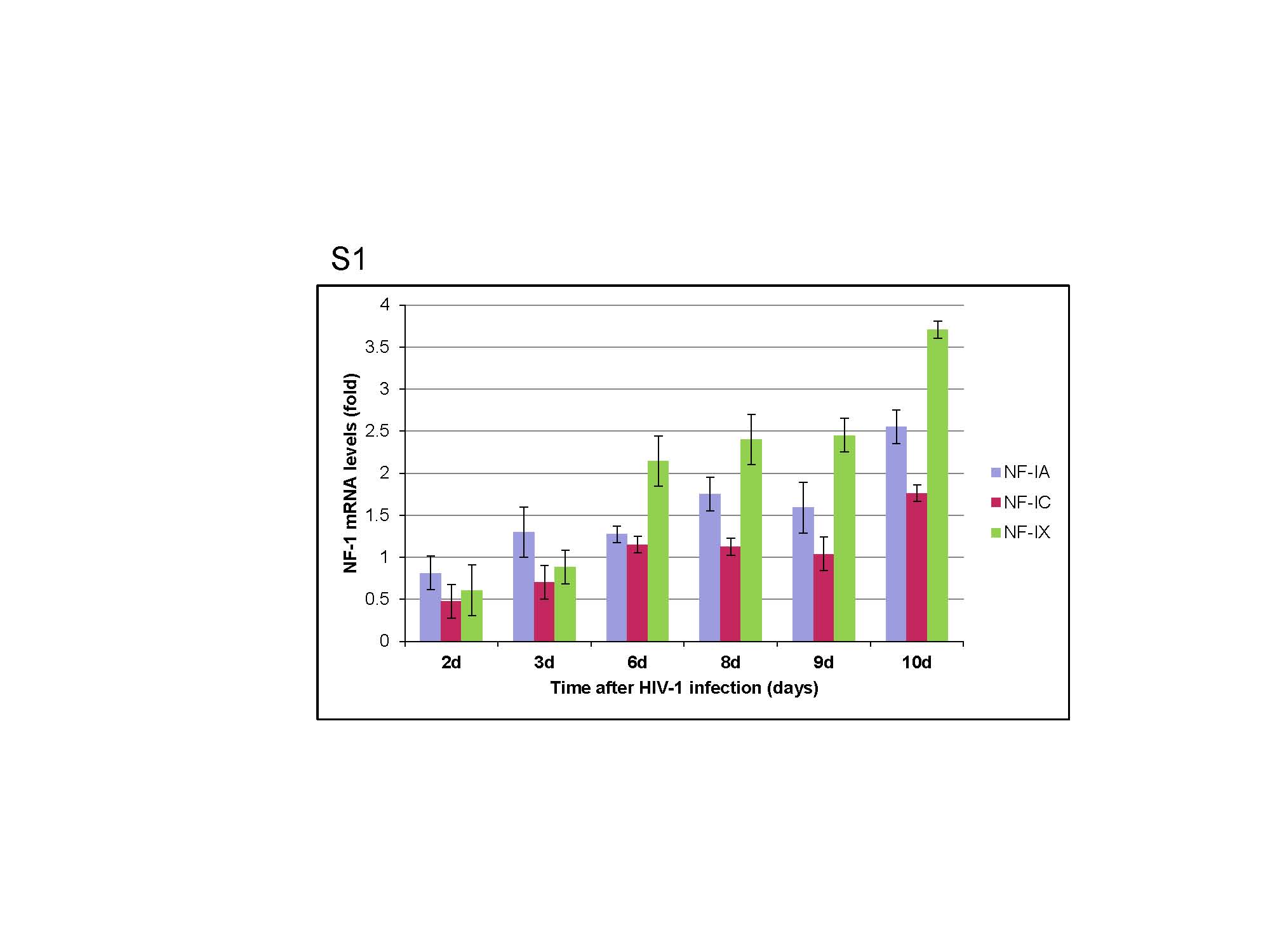
*

**Figure S1.** Expression of NF-1 family members (NF-1A, NF-1C, and NF-1X) following HIV-1 infection. Jurkat cells were infected with HIV-1 III-B strain using
5 ng equivalents of p24/10^6^cells. At the indicated time points post-infection, cells were harvested to measure expression of NF-1A, NF-1C, and NF-1X mRNA levels using real-time quantitative RT-PCR. The gene expression levels were normalized against glyceraldehyde-3-phosphate dehydrogenase (GAPDH).The data represents the mean *±*SEM from three experiments*.*

*
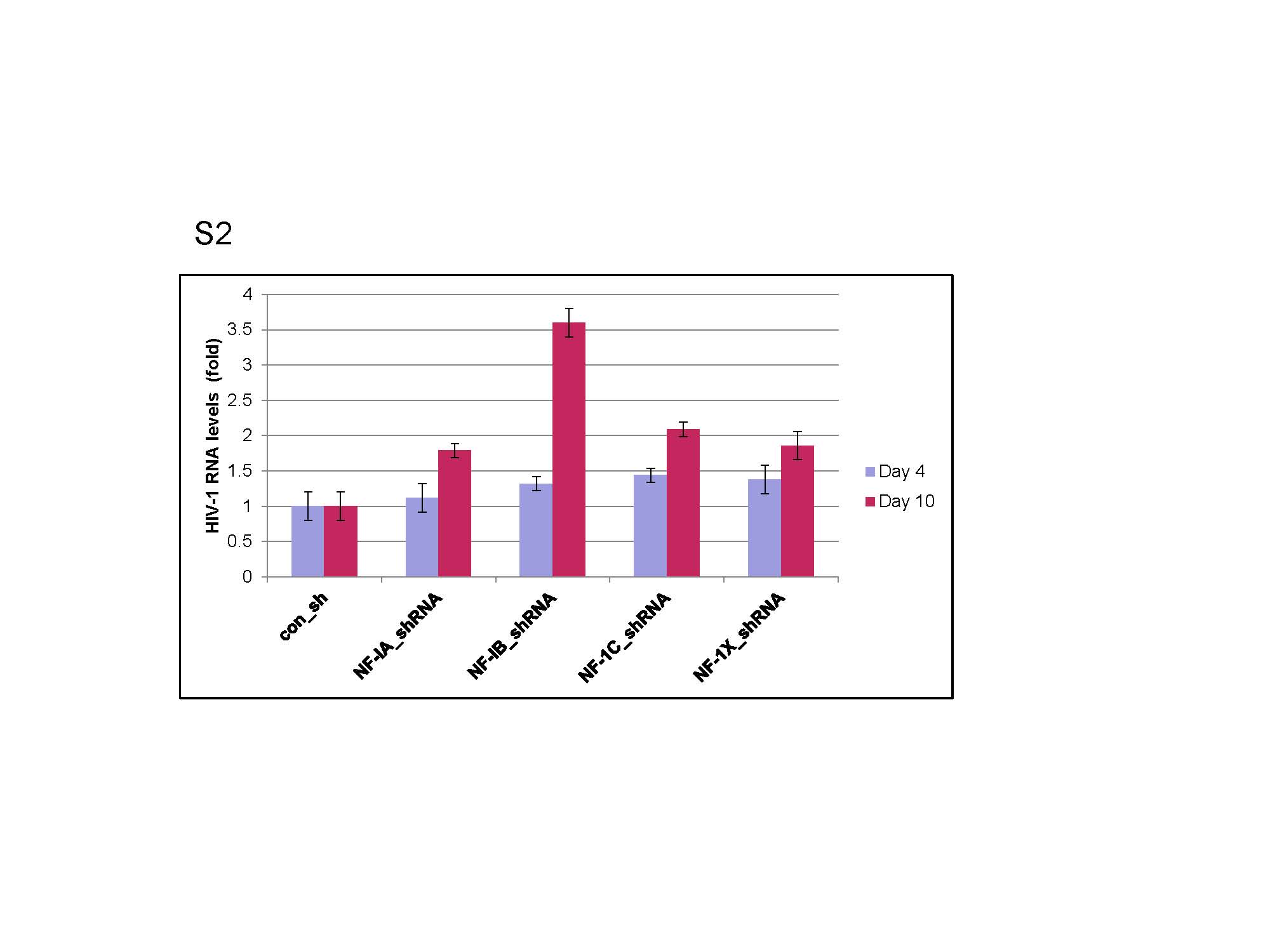
*

**Figure S2.** HIV-1 infection in NF-IA, NF-1B, NF-1C, and NF-1X knock-down Jurkat cells. Expression levels of NF-1 family members, NF-IA, NF-1B, NF-1C, and NF-1X were knocked down in Jurkat cells using MISSION shRNA lentiviral particles (Sigma, USA) targeting NF-1A, NF-1B, NF-1C and NF-1X genes. As negative controls, cells were transduced with the MISSION pLKO.1-puro Non-Target shRNA Control Transduction Particles (sh-Control) that contain an shRNA insert that does not target any human gene.
Sh-NF-I or sh-control selected Jurkat cells were infected with HIV-1 and cultured in
RPMI-1640 growth media. On days-4 and day-10 post-HIV-1 infection, cells were tested for the HIV-1 gag levels using quantitative real-time PCR. The data represent the mean *±*SEM from three experiments*.*
